# Multiplexed SNP Typing of Ancient DNA Clarifies the Origin of Andaman mtDNA Haplogroups amongst South Asian Tribal Populations

**DOI:** 10.1371/journal.pone.0000081

**Published:** 2006-12-20

**Authors:** Phillip Endicott, Mait Metspalu, Chris Stringer, Vincent Macaulay, Alan Cooper, Juan J. Sanchez

**Affiliations:** 1 Department of Zoology, University of Oxford Oxford, United Kingdom; 2 Department of Evolutionary Biology, Estonian Biocenter, University of Tartu Tartu, Estonia; 3 Natural History Museum London London, United Kingdom; 4 Department of Statistics, University of Glasgow Glasgow, United Kingdom; 5 Australian Centre for Ancient DNA, University of Adelaide Adelaide, Australia; 6 Instituto Nacional de Toxicologia, Departamento de Canarias Santa Cruz de Tenerife, Spain; University of Utah, United States of America

## Abstract

The issue of errors in genetic data sets is of growing concern, particularly in population genetics where whole genome mtDNA sequence data is coming under increased scrutiny. Multiplexed PCR reactions, combined with SNP typing, are currently under-exploited in this context, but have the potential to genotype whole populations rapidly and accurately, significantly reducing the amount of errors appearing in published data sets. To show the sensitivity of this technique for screening mtDNA genomic sequence data, 20 historic samples of the enigmatic Andaman Islanders and 12 modern samples from three Indian tribal populations (Chenchu, Lambadi and Lodha) were genotyped for 20 coding region sites after provisional haplogroup assignment with control region sequences. The genotype data from the historic samples significantly revise the topologies for the Andaman M31 and M32 mtDNA lineages by rectifying conflicts in published data sets. The new Indian data extend the distribution of the M31a lineage to South Asia, challenging previous interpretations of mtDNA phylogeography. This genetic connection between the ancestors of the Andamanese and South Asian tribal groups ∼30 kya has important implications for the debate concerning migration routes and settlement patterns of humans leaving Africa during the late Pleistocene, and indicates the need for more detailed genotyping strategies. The methodology serves as a low-cost, high-throughput model for the production and authentication of data from modern or ancient DNA, and demonstrates the value of museum collections as important records of human genetic diversity.

## Introduction

The rapidly increasing number of characterised human single nucleotide polymorphisms (SNPs) available on public databases represents a powerful resource with which to improve our understanding of population history, disease susceptibility and forensic studies[1]. Yet the conventional approach used to produce this data, PCR amplification followed by sequencing or restriction fragment length polymorphism (RFLP) mapping, is known to generate error-strewn data sets[2]–[4]. The potential for contamination or mix-ups between samples increases rapidly with the number of steps or genotyping reactions required, and the approach also requires a large amount of resources (time, reagents, template DNA).

With whole genome mtDNA data, the problem of multiple assays is exacerbated by the sheer number of nucleotides sequenced and interpreted. It is not uncommon for such data sets to contain mistakes arising through a combination of sub-optimal sequencing chemistry, automated analysis and human error[5]–[10]. Although some authors argue against whole genome sequencing as a routine approach[11], there is general agreement that mtDNA population genetics requires coding region information to both increase resolution and identify homoplasy common in control region data. With increasingly detailed phylogenetic interpretations being made from both modern[12] and ancient[13] DNA, the accuracy of both the novel and comparative genetic data is of paramount importance.

These issues are exemplified in genetic analyses of the indigenous peoples of the Andaman Islands, in the Bay of Bengal (Indian Ocean), who remain central to discussions concerning the early population history of Asia, including the concept of a ‘southern route’ for the last successful migration of modern humans out of Africa to Australia[14]–[17]. Due to their distinctive phenotype and languages the inhabitants of these islands are often considered to be physically and genetically isolated from the rest of South-East Asia. The continental shelf joining the Andaman archipelago with Myanmar is relatively shallow and there may have been the opportunity to cross by a land-bridge, or over short stretches of water during periods of depressed sea levels during the late Pleistocene.

The two main mtDNA lineages (M31 and M32) found in the Andamanese both contain control region motifs that are very common throughout haplogroup M[18]–[20], which is considered to be the broader genetic marker for an early, southern migration route of humans from the Indian sub-continent eastwards[21], [22]. As a consequence, the genetic evidence about the origins of the Andamanese[20], [23] remains ambiguous, and increased resolution is important. Recently, a sister clade of the M31 lineage has been detected amongst a tribal population in East India[24], which might indicate that M31 reached the Andamans after the first migrations of humans carrying haplogroup M through the region. However, these interpretations are dependent upon whole-genome sequences that cannot be assumed to be flawless given the number of errors identified in other mtDNA sequencing studies.

Although a proven methodology exists for retrospectively identifying errors in published mtDNA data through careful phylogenetic analysis[25]–[27] this form of internal control is often neglected, particularly when many of the SNPs reported have no bearing on the research being conducted[28], [29]. It is also heavily reliant upon comparative data that may not be available; for example, when sequencing novel mtDNA lineages such as those present in the Andaman Islands[23], or phylogenetically deep-rooting genomes. Therefore, in all situations, the primary concern of the laboratory should lie with the accuracy of data production. However, even when all reasonable safeguards are taken, the issue of how to discern genuine novel variation from sequence or human errors remains, especially when working with the degraded templates typical of ancient DNA (aDNA)[30]–[32].

Multiplexed PCR reactions provide a potential solution to these problems, because multiple primer pairs amplify different parts of the genome together in a single reaction tube[33]. Sequence variants within the multiplex PCR products can be characterised by numerous approaches[34], but the technically simplest and most reliable method of detecting specific nucleotide variation(s) appears to be single base extension (SBE) where one of four fluorescently labelled ddNTPs present is incorporated 5′ of the SBE primer and screened with a capillary sequencer[35]. The SBE technique is less sensitive to the frailties of conventional sequencing, because only a single ddNTP needs to be incorporated, and the assay can be designed to work in either direction to overcome strand-specific amplification and sequencing problems.

Despite a relatively long history[36], there have been few SNP typing studies using a multiplex methodology and the method has not yet been adopted for the authentication of data. Typically, the number of SNPs tested is small so assays often require multiple reactions to achieve acceptable levels of discrimination between samples[35], [37], [38]. Although some assays target segments over 800 bp[39], it is desirable to limit designs to the smallest possible products in order to minimize the error rate due to allelic drop out. This approach has been used in forensics studies but assays still usually target segments too large for degraded templates typical of aDNA[40]. With very well preserved ancient specimens it is possible to use a two-step multiplex PCR to generate large segments of mtDNA sequence data[41] but this involves a second round of exponential amplification for each individual product. In contrast, SBE reactions can use PCR products as small as 50–60 bp for templates, and can be multiplexed in a linear amplification, providing greater sensitivity.

The enormous demographic loss during the British colonial era makes it impossible to adequately reconstruct the evolutionary history of the remaining Andaman populations, especially those of the Greater Andamans, who are critically important because they formed the absolute majority at the time of colonisation (1858), and spoke a different set of dialects[18]. Therefore, this study utilizes the resources of human skeletal material from the colonial era preserved in various museum collections in an attempt to reconstruct the population diversity present in the latter part of the 19^th^ century. Due to the predictably degraded nature of the DNA, the key to this strategy is a PCR design which targets products under 130 bp in length, without sacrificing any of the highly specific target SNPs, through careful primer design[42].

**Figure 1:**
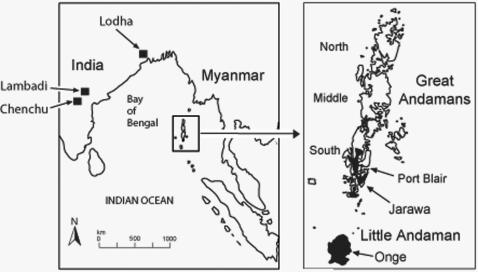
Map of the principal islands in the Andaman archipelago in the Bay of Bengal showing the major tribal divisions present in 1858. The locations of the three contemporary Indian tribal populations surveyed are given on the regional map.

Twenty potentially informative coding region SNPs were identified using whole mtDNA genome data from two previously defined mtDNA lineages, M31 and M32[20], [23], [24]. These SNPs were used to genotype 20 historical samples of Andaman Islanders from the Bay of Bengal (Fig. 1), assigned by their control region sequences to M31 and M32[23]. The samples cover three different linguistic groups; Greater Andamanese (n = 16), Jarawa (n = 2), and Onge (n = 2). Because two of the M31 SNPs are present amongst the Rajbansi of West Bengal (forming a sister clade M31b[24]) the multiplex method was also used to screen modern DNA extracts from three Indian tribal populations (Chenchu, Lambadi, and Lodha), who possess similar control region motifs. Late Pleistocene inter-regional mtDNA links are the exception in South and South-East Asia[21], [43], so any genetic connections would be of considerable importance to reconstructing human migration and settlement patterns during this formative period.

## Results and Discussion

**Figure 2:**
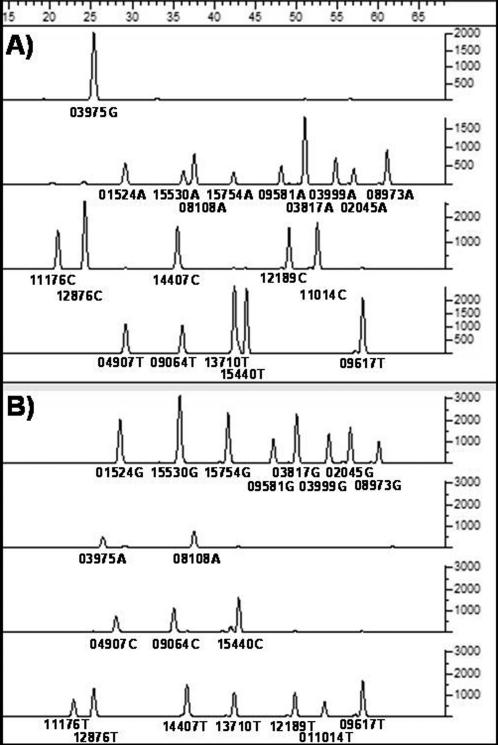
Genotypes of two Andaman Islanders using a panel of 20 informative SNPs. Each line represents the four systems of G, A, C, and T, in descending order. SNPs are named by their position relative to the Cambridge Reference Sequence and are separated by variation in electrophoretic mobility, which is determined by the length of the SBE primer. The segregation of the two samples at all lineage defining sites, for M31a1 and M32 respectively, demonstrates the accuracy and reliability of genotyping using this methodology.

**Figure 3:**
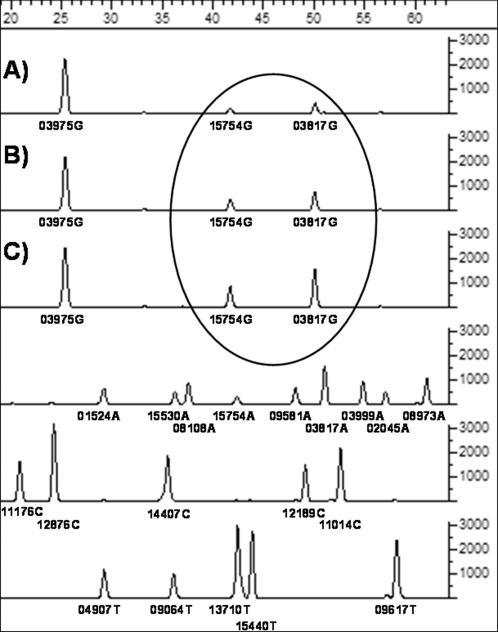
The results from a Snapshot assay of an M32 sample that has been deliberately contaminated with a modern European DNA extract. Alleles at np15754 and 3817 on the G line are from the contaminant DNA, whilst those on the A system represent the M32 sample. The level of the contaminant increases, as a proportion of the total mix, with each of the descending lines shown within the oval. The relative amounts of contaminant are 4%, 8% and 20% of the effective copy number (assessed by quantitative PCR) prior to mixing of the two extracts to seed the multiplex.

The final cross-validated results of the SBE amplifications are presented in Table 1, and show that all target SNPs were determined for each sample, demonstrating the robustness of the multiplex methodology. Fig. 2 shows the actual profile of two samples, one each from the M31a and M32 mtDNA lineages. The M31 and M32 genotypes are distinctive, aiding the identification of contaminating DNA[32], but the behaviour of multiplexed SBE reactions in the presence of multiple populations of templates is not well studied. So, to further investigate the specificity and sensitivity of the multiplex-SBE genotyping methodology one of the Andaman M32 samples was mixed with decreasing amounts of a modern European extract, which had been diluted to the same concentration, and the resulting aliquots were used to initiate the M31_M32 multiplexed SBE assay. The presence of both alleles at each of the M32 specific sites (Fig. 3) indicates the presence of the second set of (contaminating) templates, which are detectable at all concentrations. The lowest of these is just 4%, a level that is beyond the threshold of detection by routine cloning approaches[44].

The absence of additional alleles at any of the segregating sites for all twenty samples in Table 1 indicates that contamination, either from endogenous sources or between samples, did not affect the results. Yet, the multiplex reveals that the A08108G mutation occurs in both M31 and M32. Although this SNP is also present in the East Asian mtDNA haplogroup M11[45], it would normally be considered unusual to find it on two independent lineages in the same geographic location. If a standard singleplex PCR approach had been used, there would be a strong suspicion of artificial recombination via sample mix up. However, the simultaneous amplification of all the SNPs excludes this possibility because only single alleles have been observed and all other sites tested are consistent.

**Figure 4:**
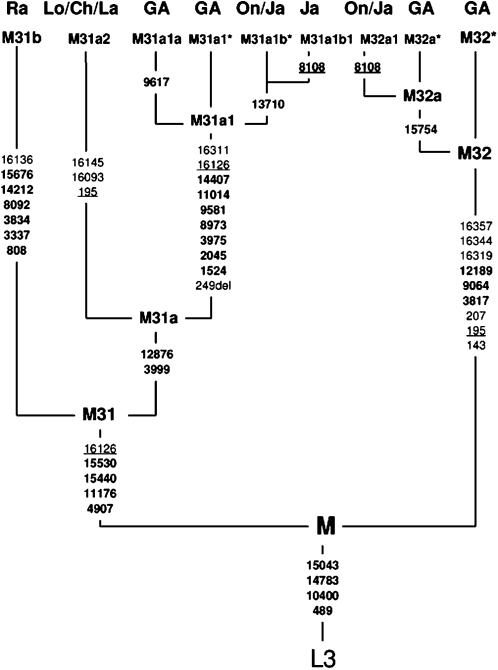
Maximum parsimony tree of mtDNA lineages M31 and M32 obtained by multiplexed genotyping, demonstrating the fine structure apportioned to these lineages [Ra = Rajbanshi; Lo = Lodha; La = Lambadi; Ch = Chenchu; GA = Greater Andamanese; On = Onge; J = Jarawa]. The definitions of the Andaman-specific M31a1 and M32 lineages are revised to include additional control region SNPs resulting from the cloned sequences of the historic samples. The Indian variant found amongst the Lodha, Chenchu and Lambadi develops the inter-regional structure of M31 and allows for the settlement of the Andaman archipelago substantially after the last successful migrations Out of Africa by modern humans. These sequences contribute a deletion at np249 on the trunk of M31a1 and the motif of 143-195-207 on the stem of M32, contra to the tree of Thangaraj et al.[23], which did not report the deletion and the three M32 SNPs never occurred together in their five samples. The original data also reported a transition at np200 on all four of their M31 samples, but in the historic data it is only found in a single Onge sample.

**Figure 5:**
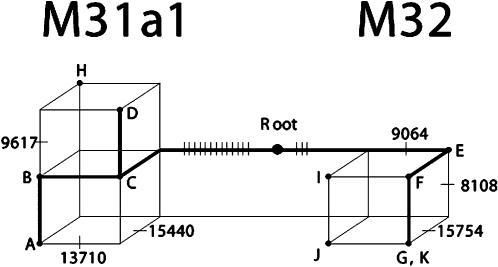
Full median network depicting the conflicts between the data sets of this study and that of Thangaraj et al.[23] in the coding region Nodes A-K represent distinct haplotypes. A-G correspond to our hapolotypes assigned to M31a1b1, M31a1b*, M31a1*, M31a1a, M32*, M31a* and M32a1, respectively, whilst H-K are from Thangaraj et al[23]. The node marked “Root” is the root of haplogroup M and ticks on the network indicate mutations, the most important of which are shown by their position; the long internal branches are shown compressed. The diagram summarizes all most parsimonious reconstructions of mutations on a tree. Note how our data can be explained (bold) by a single homoplasy at position 8108. However to attach H, I and J to the bold tree would require a further four recurrent mutations to be introduced.

The revised phylogenetic structure of M31 and M32 is depicted in Fig. 4, which includes data from the control region sequences provided in the supplementary information (Table S4). Both lineages now have substantial internal structure and revised groups of defining mutations. Of particular interest is the establishment of the major clade M31a, which is present in both South and South-East Asia, linking the two regions more recently than previously thought. The main conflicts in the coding-region data between this tree and the previous data sets, together with the resulting reticulations are depicted in the network presented in Fig. 5. The thicker line represents the most parsimonious phylogeny for the M31a1 and M32 mtDNA lineages.

**Table 1:**
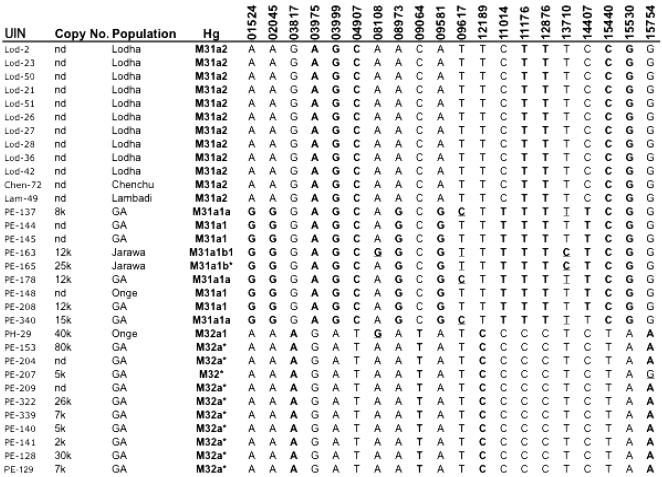
Results from the M31-M32 multiplex.

The genotyping results demonstrate that the errors occurring in published mtDNA data sets can be much more than mere ‘background noise’. The correction of errors and the discovery of novel mutations have significantly altered the M31 and M32 phylogenies, providing important additional fine structure. Of special significance are the two sub-clades of M31a1 defined by A90617G (M31a1a) and A13710G (M31a1b), which are separated both geographically and culturally, being broadly contiguous with speakers of the Greater Andamanese (GA) and Onge-Jarawa (On/Ja) dialects, respectively. A similar pattern is apparent in M32, where M32a1 is only found amongst the Onge-Jarawa populations. This intriguing result, suggests a high degree of genetic as well as cultural isolation within the Andaman Islands.

Until the modern data sets are corrected, the sample sizes for Onge and Jarawa limit what can be said about genetic input to these populations. However, it is difficult to reconcile the lack of overlapping haplotypes amongst the Greater Andamanese simply as a product of genetic drift, unless there was an extremely limited movement of women from the neighbouring Jarawa for a substantial period of time. This finding is somewhat surprising given that these two groups shared a common border. The clear structuring mirrors observations from linguistics, which place the Onge and Jarawa languages together[46], and material culture where different bow types reflect the same divisions[47]. The length of this isolation is difficult to determine exactly but assuming a mutation rate of one new coding region SNP per 5300 years[48] this would place it somewhere during the last 10,000 years, and could, therefore, be associated with the rising sea levels of this period.

These important insights into prehistoric demography were made possible by the recovery of information from the Greater Andaman populations and demonstrate the potential of museum collections for reconstructing the genetic diversity of pre-colonial and early colonial populations. The sensitivity of the SBE reaction and its ability to detect multiple populations of templates indicate that these collections do not suffer from routine contamination by exogenous DNA. This demonstrates that repeated handling and washing is not necessarily the source of contamination in DNA extractions from human teeth[49], despite the Andaman Islanders being one of the most studied sets of human remains held in European museums. It also shows that clean extractions from human skeletal material are possible provided adequate care and suitable precautions are taken in the laboratory.

The rapid screening potential of the multiplex for specific mtDNA lineages is exemplified by the characterisation of M31a2 amongst the Chenchu, Lambadi and Lodha, and highlights the limitations of the control region for phylogenetic analysis. This is because M31a1 and M31a2 have completely different motifs, and the latter appears (erroneously) to be linked to M31b. If the analysis had been limited to control region sequences the connection would never have been made, and it is difficult to see how it could have been detected without the availability of whole-genome data[11].

This inter-regional connection is significant because it demonstrates a shared maternal gene-pool approximately half the age of haplogroup M31 itself. Assuming an age for haplogroup M of around 60 k years[48] the six coding region mutations preceding the splitting of M31a would centre this date around 30 kya. This is close to LGM low sea levels (20–25 kya), which may be significant in terms of access to the archipelago from the mainland. Without comprehensive data from Myanmar it is not possible to identify whether the Andaman M31a1 arrived from India or if the Indian M31a2 came from South-East Asia. But either scenario casts serious doubts on the concept that the Andaman Islands were settled at the time of the migrations out of Africa carrying the current Eurasian mtDNA diversity.

The additional insights into this seminal period of human pre-history afforded by this study suggest that more comprehensive sampling of contemporary populations is required and that tribal groups may be an important reservoir of genetic diversity vital to reconstructing human evolutionary history. In the context of the current study, Myanmar may prove vital to retracing population movements of the Late Pleistocene, including the settlement of the Andaman archipelago. It is also clear that there is an important role for museum collections in this process to recover crucial signals from populations that did not survive their encounter with modernity, or who have suffered considerable demographic reduction.

Whether using modern or ancient samples, the level of resolution required for this work necessitates the availability of whole-genome sequence data and a method for the rapid genotyping of individuals, but this is itself dependent on the sequence data being correct.

The levels of accuracy, built-in controls for internal consistency (particularly useful for aDNA), and a reduced need for reagents and templates make multiplexing with SBE an ideal tool for detailed population phylogenetics with both ancient and modern human DNA. The incorporation of this methodology into the protocols of laboratories would also lead to a significant reduction of errors prior to data publication. In a more general study, a lower resolution multiplex design could quickly assign individuals to the major mtDNA haplogroups, establishing the correct phylogenetic placement and identifying novel or related lineages for further investigation via whole genome sequencing.

Once a framework is established it is simple to design a multiplex to check sequence variations for the early identification of errors. This methodology would allow additional population samples to be rapidly checked, further reducing the chance of mistakes entering the public databases, and has the potential to uncover fine structure within the data. The initial cost of developing multiplex assays is quickly repaid in savings of both time and reagents due to the parallel testing of SNPs. The simple protocols described here mean that this powerful experimental procedure is within the reach of all laboratories currently producing direct sequence data.

## Materials and Methods

DNA was extracted from powder removed from the dental cavity of teeth held in the collections of the Natural History Museum London, the Oxford University Museum of Natural History, and the Royal College of Surgeons Edinburgh. Tooth external surfaces were cleaned by wiping with sodium hypochlorite solution (6% w/v) before being encased in dental alginate; this facilitates specimen handling while also preventing exposure of the outer contaminated surfaces to either the laboratory or personnel. A dental motor and 90 degree hand-piece (Bien Aire, Switzerland) were used to slice through the alginate-encased roots, and remove the surface layers of the exposed dental cavity. Thirty milligrams of powder was digested on a rotor for 20 hours at 55°C (50 mM EDTA; 10 mM ea Tris-HCL ph 8.0, NaCl, N-phenacylthiazone bromide; 20 mM proteinase K, 80 mM dithiothreitol, and 1% w/v sodium dodecylsulphate), followed by a standard Phenol-Chloroform extraction[50] (2 Phenol washes, 1 Chloroform), and washed by H_2_0 (twice) on a Microconcentrator (30,000KD, Millipore USA), to generate a final volume of 125 µl (for details of the modern samples see Kivisild et al.[51] and Metspalu et al.[21]).

The Henry Wellcome Ancient Biomolecules Centre (ABC) at Oxford was used to extract DNA, and set up PCRs. The ABC is physically isolated, subject to stringent anti-contamination procedures, equipped with positive air pressure and UV lighting, and has a DNA laboratory and equipment (glove box, instruments, full body suits, protective masks, etc.) dedicated solely to ancient human specimens. The thermal cycling reactions and subsequent downstream work took place in a separate laboratory located in the Department of Zoology. Quantitative PCR (qPCR) was used to measure amplifiable copy number and identify inhibition in a subset of the extracts, using SYBR Green® master-mix (AB), human-specific primers synthesized standards (Biomers, Germany) and an ABI 7000 real-time PCR machine. The results were used to calculate dilution and loading levels of template DNA (Table S4). For the samples analyzed, an effective copy number of 10 k–20 k molecules per µl produced optimal results, although no reduction in fidelity was observed with copy number as low as 2 k per µl.

PCR primers were designed to give product lengths ranging from 67 to 127 nucleotides (Table S1). The aim was to obtain a theoretical melting temperature of 60°C±2°C and a purine:pyrimidine content close to 1∶1 using Primer3 (http://www-genome.wi.mit.edu/cgi-bin/primer/primer3_www.cgi). Primer candidates were checked for primer-dimer formation, hairpin structures, homology and complementarity to other primers in the multiplex according to the guidelines outlined in[33]. All oligonucleotides were synthesized and reverse-phase HPLC purified by Biomers.net GmbH (Ulm, Germany). The initial multiplex PCR was performed in a 25 µl reaction volume containing 1× PCR buffer, 6.5 mM MgCl_2_, 600 µM of each dNTP, 0.01 µM of each primer, and 2 U of AmpliTaq Gold® DNA polymerase (AB) using the following cycling programme: denaturation at 94°C for 5 minutes followed by 35 cycles at 95°C for 30 sec., 60°C for 30 sec., and 65°C for 30 sec., followed by 6 minutes at 65°C. 2.5 µl of PCR products were purified by adding 0.75 units Shrimp Alkaline Phosphatase (SAP, Amersham Biosciences) and 0.225 units Exonuclease I (ExoI, USB Corporation). The mix was incubated at 37°C for 30 minutes, and inactivated at 75°C for 15 minutes.

The SBE primers were designed to range between 16 and 60 nucleotides, using size intervals of 4 nucleotides for primers longer than 32 nt nucleotides and 5–7 nucleotides for shorter primers to allow for differing electrophoretic mobility (Table S2). Loci with opposite allele combinations (e.g. A/G with C/T) were analysed with SBE oligos in the same size interval whenever possible, to maximize the number of SNPs typed in the minimum read window. SBE reactions were performed in a total volume of 5 µl with 0.5 µl purified PCR product, 2.5 µl SNaPshot™ reaction mix (AB) and 0.5 µl SBE primer mix (0.05 µM each primer and 300 mM Ammonium Sulphate), using the following program: 35 cycles of 96°C for 10 sec., 50°C for 5 sec., and 60°C for 30 sec. After the SBE reaction, 1U of SAP (Amersham Biosciences) was used as above. One µl of SAP purified SBE product was then mixed with 20 µl Hi-Di formamide (AB) and analyzed by capillary electrophoresis using an ABI 3100 genetic analyzer with 50-cm capillary arrays, POP-6 polymer (AB), injecting for 22 seconds at 3.0 kV and a run-time of 25 min. Negative controls were included in both stages of the assay to assess the potential for cross-contamination between samples and amplification of non-Andamanese DNA.

Automated allele calls were made using macros constructed in Genotyper 3.7 (AB). All peaks in the size standard were detected and the height of the largest peak in the electropherogram had to be a minimum of 800 Relative Fluorescent Units before analysis could proceed. Adjustments were made to the pre-defined windows allowing for variation in electrophoretic mobility, unequal emission energies and differential incorporation of the fluorescent ddNTPs[52], [53]. Primer concentrations were adjusted to give balanced amounts of products and peak heights, concentrating on the lineal SBE reaction[54].

Once all peaks were reliably and reproducibly called, SNP calls that did not conform to those expected from the main phylogenetic tree of Thangaraj et al.[20], [23] were re-assessed using singleplex SBE reactions to exclude the possibility of interference from other sites, and all found to be accurate. An additional cross-check was performed by re-amplifying the original multiplex products (1 µl of PCR product diluted 1∶100 in a 25 µl volume reaction: 1× buffer, 2 mM MgCl_2_, 250 µM dNTPs, 0.25 U Taq, 250 nM each primer; at 94°C for 2 minutes followed by 30 cycles at 94°C for 45 secs., 58°C for 45 sec., and 72°C for 90 sec., followed by 10 minutes at 72°C), before gel purification and cloning with TOPO TA Cloning® kit (Invitrogen, UK) as per the supplier's instructions. Eight white colonies were amplified using T7 and M13R primers, and sequenced using ABI BigDye® Terminator v3.1 and an ABI Prism® capillary DNA 3700 automated sequencer. The same protocols were used for the production of control region sequences using additional pairs of primers designed to the same specification as those in the multiplex (Table S3). All PCR products and negative controls were checked by electrophoresis on 3% agarose gels, prior to purification with a Montage® PCRµ96 system (Millipore, U.S.A).

Independent replication of the results was not undertaken for this study because it was considered that sufficient grounds are provided for accepting the results as they stand[55]. The absence of any allelic dropout, or detectable contamination, in the twenty-plex SBE reaction provides a novel method of judging the accuracy of the results, and demonstrates the extracts are free of non M31_M32 DNA. The multiplex assays were repeated, without variation, up to four times for each sample, and real-time quantitative PCR reported copy number consistent with the ability to repeat the multiplex results. The control region haplotypes (also multiplexed and re-amplified from the same DNA aliquot) are 100% consistent with the two Andaman specific haplgroups assigned by the multiplex SBE results. These sequences are invariant at all the main hg defining sites, and additional control region diversity (Table S4) is limited to two nucleotide positions and one of these (np 16129) is found in three different samples. All deviations from the published data[23] were rigorously cloned and showed no ambiguity at the sites tested.

## Supporting Information

Table S1 Details of M31_M32 multiplex PCR primers and target SNPs
(0.41 MB EPS)

Table S2 Details of M31_M32 SBE primers
(2.25 MB EPS)

Table S3 Details of PCR primers for control region sequencing
(0.33 MB EPS)

Table S4 Details of control regions polymorphisms detected in each sample.
(1.72 MB EPS)
